# Lateral Motion Prediction of On-Road Preceding Vehicles: A Data-Driven Approach

**DOI:** 10.3390/s19092111

**Published:** 2019-05-07

**Authors:** Chen Wang, Jacques Delport, Yan Wang

**Affiliations:** 1 The Bradley Department of Electrical and Computer Engineering, Virginia Polytechnic Institute and State University, Blacksburg, VA 24061, USA; 2 The California ISO, 250 Outcropping Way, Folsom, CA 95630, USA; jdelport@caiso.com; 3 Department of Urban and Regional Planning, Florida Institute for Built Environment Resilience, University of Florida, Gainesville, FL 32611, USA; yanw@ufl.edu

**Keywords:** data-driven intelligent vehicles, data mining, driver behavior classification, lateral motion prediction, vehicle mobility data

## Abstract

Drivers’ behaviors and decision making on the road directly affect the safety of themselves, other drivers, and pedestrians. However, as distinct entities, people cannot predict the motions of surrounding vehicles and they have difficulty in performing safe reactionary driving maneuvers in a short time period. To overcome the limitations of making an immediate prediction, in this work, we propose a two-stage data-driven approach: classifying driving patterns of on-road surrounding vehicles using the Gaussian mixture models (GMM); and predicting vehicles’ short-term lateral motions (i.e., left/right turn and left/right lane change) based on real-world vehicle mobility data, provided by the U.S. Department of Transportation, with different ensemble decision trees. We considered several important kinetic features and higher order kinematic variables. The research results of our proposed approach demonstrate the effectiveness of pattern classification and on-road lateral motion prediction. This methodology framework has the potential to be incorporated into current data-driven collision warning systems, to enable more practical on-road preprocessing in intelligent vehicles, and to be applied in autopilot-driving scenarios.

## 1. Introduction

According to the latest survey about causes of motor vehicle crashes in the United States [1], nearly 94 percent of crashes in 2015 were attributed to driver-related errors, including recognition, decision, performance, and non-performance errors. Approximately 33 percent of crashes caused by driver-related errors were due to the false prediction of actions by others, misjudgment of vehicle distance and speed, and other decision errors, while recognition errors caused by driver inattention and inadequate surveillance were responsible for 41 percent of these accidents alone. The false prediction of lateral motions by other vehicles is exceptionally dangerous for both the ego vehicle and the preceding ones. This maneuver is one of the riskiest movements during driving due to its changes in both the longitudinal and lateral velocity in the presence of the surrounding moving vehicles [2,3]. Nevertheless, successful prediction can allow the driver more certainty to prepare for reactions. In addition to the basic safety concerns, the development of the advanced driver assistance systems (ADAS) in future intelligent driving systems also requires this predicting scheme to resolve the uncertain behaviors of human drivers in order that they can co-exist within the foreseeable future.

With the rapid development of data-driven intelligent transportation system [4], there is an increasing number of studies using data from multiple sources (e.g., inductive loop detectors, laser radar, GPSs, and other in-vehicle data acquisition devices) to provide timely and accurate transportation and driving information for on-road drivers. However, the development of the auto-driving vehicles based on these advanced sensing technologies is a process and there will be a stage when auto-driving and the human driver co-exist. The behaviors of human drivers are unpredictable compared to pre-programmed driving assistance systems. It is necessary to develop a mechanism for the program-based auto-driving system to predict possible actions of human drivers and to be prepared to respond to the possible lateral motions of the preceding vehicles. With the proposed prediction method, the driving assistance system on the ego vehicle can utilize the mobility data of the preceding vehicles, which is acquired from the sensors installed, to classify the driving manners of the human driving the preceding vehicle and further use machine learning methods to estimate their behaviors. With this knowledge, the safety of the ego vehicle can be improved by reducing the uncertainty caused by other surrounding vehicles.

Therefore, we propose a two-stage, data-driven approach to predict the lateral motions of the preceding vehicles. The Gaussian Mixture Model (GMM) is used to classify the driving behavior into erratic and consistent. This result is then utilized to bias the lateral motion predictions made by two types of ensemble decision trees for more safety. To implement the proposed methodology, we utilize real-world vehicle mobility data acquired from Ann Arbor, Michigan, provided by the U.S. Department of Transportation (USDOT) [5]. The proposed prediction methodology of surrounding vehicles has the potential to be integrated into autonomous or assistance-based driving systems. Its functionality can assist drivers to make decisions and to increase safety. 

This paper is organized as follows: In Section 2, several relevant works are reviewed, and the goal of this paper is proposed with the research gap. In Section 3, the real-world data used for the algorithms’ training is introduced, and data preprocessing techniques are described. In Section 4, the methodology is proposed, and some initial prediction results are presented. In Section 5, the test results of the trained prediction model based on a larger set of data are listed and discussed. Finally, Section 6 concludes the paper.

## 2. Literature Review

### 2.1. Intelligent Driving System and Vehicle Motion Prediction

Currently, there are three types of models for vehicle motion prediction: physics-based motion, maneuver-based motion, and interaction-aware motion predictions [6]. The physics-based motion models consider vehicles as dynamic entities following the laws of physics. The maneuver-based motion models take the maneuvering behaviors of the driver into consideration when evaluating vehicles actions. These maneuvers are considered as independent. The interaction-aware motion model represents vehicles as maneuver entities interacting with each other, i.e., the influence brought by the motion of other vehicles is contemplated. The prediction in this paper is focused mainly on vehicle kinetic statuses. These statuses are the result of the behaviors of drivers, especially maneuver actions. Therefore, our proposed model is related to both physical-based and maneuver-based motion prediction. There are multiple existing researchers using similar types of models. Hsu et al. (2012) utilized a sensor fusion system composed of a three-antenna GPS, four suspension displacement sensors, and an inertial measurement unit to acquire real-time six-degree-of-freedom vehicle dynamics. The dynamic data are used to estimate the system dynamic model based on the recursive least squares estimation method. The future dynamics of the vehicle are predicted using this model based on the real-time data fed by the sensors [7]. Sekizawa et al., in 2007 developed a stochastic switched autoregressive exogenous (SS-ARX) model to predict the collision avoidance behavior of drivers using simulated driving data in a virtual reality system [8]. Chen et al., in 2018, designed a visibility-based collision warning system to use the neural network to reach four models to predict vehicle rear-end collision under a low visibility environment [9]. Specifically, Shan et al., in 2013, proposed a long-term vehicle position tracking and prediction model that incorporates vehicle behaviors and physical features of the driving environment (i.e., road segments, and intersections and areas). The multiple model approach used the particle filter for estimating position in the external environment [10]. Taniguchi et al., in 2015, proposed a double articulation analyzer with temporal prediction (DAA-TP) to model driving behaviors and predict their actions over time [11]. With historical traffic data, Jiang and Fei, in 2016, employed neural network models to predict average traffic speeds of road segments and a forward-backward algorithm on Hidden Markov models to predict speeds of an individual vehicle [12]. Many proposed approaches have also addressed distinct scenarios, such as crossings or highway exits. 

Nonetheless, most of these predictions have taken driver behavior as inputs, and many predictions are made based on either mathematical modeling or an isolated lab created scenario instead of using real-world context. While mathematical models can provide the most complete data, they have the limitation of modeling. Regardless of the sophistication of the model created, the real-world or real-human behavior is more complex. Because of the inevitable assumptions made there will be some information lost during the model construction. Lab experiments overcome this as they collect data of real human drivers. However, they are limited due to simulation tools and an isolated environment. The designated vehicles and lack of diversity of the drivers can also easily bias the data acquired. Therefore, data collected in a real-world context from a diverse vehicle and driver set are more representative for driving behavior studies.

### 2.2. Prediction of Vehicle’s Maneuver-Based Motion

A maneuver is a procedure or method of working that involves expert physical movement, and it is assumed that a vehicle’s future motion will match the intended maneuver of a driver [6]. Therefore, studies regarding vehicle’s motion prediction and driver intention estimation have both been included in this section. Specifically, the estimated maneuvers may include left/right turn, left/right lane change, lane keeping, braking, keep speed, safe errant or complaint violating [8,10,11,12,13]. Features that have been utilized to make the predictions include physical metrics of the vehicle (e.g., longitudinal motions metrics, and lateral motions metrics), environmental data (e.g., road structure), and driver behavior.

We are particularly interested in the real-time on-road vehicle lateral motion prediction. Although significant efforts have been made by building mathematical models and conducting laboratory experiments [14,15,16], the actual environment on the road is much more complicated, which creates remarkable differences. Therefore, lab simulations can provide limited references for on-road motions. Fortunately, statistical prediction models with more available large-scale traffic data have gained increased attention to address the method gap.

Among the proposed statistical methods, a few data-driven approaches have been applied in classifying driving patterns (e.g. car-following behaviors [13]) and predicting maneuver intention of vehicles in complex scenarios. For example, Morris, et al., in 2011, constructed a real-time on-road prediction system to detect the lane-change intention of a driver. The system employed a Bayesian extension of support vector machines (SVM), called a relevance vector machine to classify intention based on features from radars, cameras, and sensors. Yao et al., in 2013, developed a parametric lane change trajectory prediction approach based on real human lane change data. This method generated a similar parametric trajectory according to the k-Nearest real lane change instances [15]. Kumar et al., in 2013, proposed an online learning-based approach to predict lane change intention, which incorporated support vector machine (SVM) and Bayesian filtering [16]. The prediction was based on the information about the position of the ego vehicle to the road collected by lane trackers. One single vehicle with two drivers was used for the lane changing data collection and 139 lane changes were collected. The results showed that the proposed approach is able to predict the intention of a driver to change lanes on average 1.3 s in advance, with a maximum prediction horizon of 3.29 s. 

Liebner et al., in 2013, developed a prediction approach for lateral motion (i.e., going straight and turning right) at urban intersections with and without the presence of preceding vehicles [17]. The study focused on the parameter of the longitudinal velocity and the appearance of preceding vehicles. Butakov and Ioannou, in 2015, introduced a model of the kinematic characteristics of the ego vehicle before and during lane change behavior [3]. Yoon and Kum, in 2016, proposed a multilayer perceptron approach to predict the probability of lane changes by surrounding vehicles (i.e., the leftmost lane, the center lane, and the right lane) and trajectories based on the history of the vehicles’ position and their current positions [14]. Nilsson et al., in 2017, designed a lane change maneuver algorithm to estimate the existence of a longitudinal trajectory that allows the lateral motion of a lane change maneuver [2]. Woo et al., in 2017, constructed a lane change prediction method for surrounding vehicles. The method employed SVM to classify driver intention classes based on a feature vector and used the potential field method to predict trajectory. Experimental results demonstrated that the approach could detect a lane change 1.74 s before the target vehicle crossed the centerline with 98.1% accuracy [18].

Overall, in Section 2.1 and Section 2.2 the aforementioned studies on maneuver-based motion predictions are mostly based on laboratory experiments and very few used real-world data. Most on-road real-time prediction approaches have high requirements for data quality and need specific kinds of data to make predictions, increasing the difficulty of being utilized in driving assistant systems. The lack of information in experimental or synthetic raw data serves as an obstacle in both the general intelligent driving system design and more specific vehicle maneuver-based motion prediction. Therefore, we propose a new data-driven approach using machine learning algorithms to predict the lateral motion of the surrounding cars based on real-world vehicle data in real time. The real-world data were collected for model training with a considerable number of vehicles over a long-term period. New features were also created during a preprocessing stage to include the behavior characteristics of drivers. Therefore, the data were more informative and the model trained was more accustomed to scenarios in the real world. The functionality can be easily deployed in the vehicles equipped with any level of driving assistant systems. Only basic detection devices are required as the prediction model was trained with observations of vehicle kinematic status.

## 3. Dataset Description and Preprocessing

The dataset utilized in this paper is generated based on the safety pilot model deployment (SPMD) program [5]. As a part of the connected vehicle safety pilot program held by the USDOT, the SMPD serves as a comprehensive data repository that contains real-time vehicle mobility data, vehicle-to-vehicle (V2V) communication data, and road environment data. All the data were collected from on-road vehicles in real time with the frequency of 10 Hz to record motions, environment, and consecutive events of vehicle movements. Data were acquired from the Ann Arbor, Michigan transportation network. The periods of data collection included two separate months: October 2012 and April 2013. It was assumed that there was no large or fundamental variation in the behavior and manners of the drivers, as well as the environment, for vehicles driving within the 6-year period between the time when the dataset was collected and the time this study was conducted. On the other hand, the proposed method in this paper is adaptive to other scenarios. That being said, it is convenient for the proposed method to be utilized in any other suitable data that can provide the required information.

### 3.1. Description of the Safety Pilot Model Deployment Dataset

The SPMD dataset was composed of four parts: driving dataset, basic safety message (BSM) dataset, roadside equipment (RSE) dataset, and contextual data dataset. The driving dataset contained the vehicles’ mobility data collected by two different sets of data acquisition devices developed by the University of Michigan Transportation Research Institute (UMTRI) and the Virginia Tech Transportation Institute (VTTI), respectively. These mobility data were time-stamped and included the acquisition device ID, which was unique for each vehicle. The trips of the monitored vehicles were also identified by the status of ignition, meaning that one trip started when the ignition was on and ended when it was off. The system recorded the vehicle’s motion data, such as GPS location (longitude and latitude), speed, acceleration, and body angle change; hardware status data, such as brake status and turn signals statuses; road environment data, such as distances to the lane lines, distances and speed to surrounding objectives, and whether the vehicle had crossed the lane lines or not.

In this paper, we selected the dataset collected by the VTTI devices due to the consistency of the vehicles and their detailed spatial-temporal records. The dataset was composed of two major parts: the vehicle primary mobility data and the radar data. The primary mobility data contained motion and on-vehicle devices status records of the concerned vehicles, including 64 devices/vehicles and 14,315 trips in 61 days. The detailed data features can be found in [5], which also served as original features for model training. The radar data contained objective or target information about vehicles or objects surrounding the vehicle.

### 3.2. Time Series Data Processing

To process instances of lateral motion, the time series data have to be converted to features and labels to be acceptable to the regular data mining methods. The conversion is not meant to bring any value change to the original data. The operations involved are basically reshaping and new feature creation based on the original data. In order to do this, we found all the time tags where a lane change started to occur. We then calculated the lateral motion duration and considered a minimum time between lateral motion events in order to filter out potential mislabeling of the same lateral motion event as multiple events. For each event’s features, five-second windows (50 times tamps) were used before the turn. Negative labels were then created by chunking the rest of the data into five-second windows. Since we aimed to predict the lateral motions of the vehicle ahead and assist the driver in making a correct judgment, there should be some reaction buffer time left for the driver or the autonomous driving system. Two buffer times, m=5 (0.5 s) and m=1 (0.1 s) were deployed in the test results section to demonstrate the algorithm capability. The data containing clear lateral motion signal were eliminated, so were the data lay in the defined reaction time for the driver. The typical data chunk of positive events can be shown as in Equation (1).

(1) $$D^{\left(t\right)}=\left[\begin{array}{ccc} x_{t-n}^{\left(1\right)}, & \cdots , & x_{t-n}^{\left(d\right)}\\ x_{t-(n-1)}^{\left(1\right)}, & \cdots , & x_{t-(n-1)}^{\left(d\right)}\\ & \vdots & \\ x_{t-m}^{\left(1\right)}, & \cdots , & x_{t-m}^{\left(d\right)} \end{array}\right]$$

where, $D^{\left(t\right)}\in R^{\left(m-n+1\right)\times d}$ is the data chunk for the lateral motion event starting at time t; $x_{j}^{\left(i\right)}$ is the i-th element value at the timestamp j, i∈{1,…,d}; d is the number of elements; m is the number of timestamps ahead of the lateral motion, namely the reaction time; n=m+window_size determines the beginning time of the current data chunk.

Given the nature of the driving, this dataset is heavily skewed toward non-turning events. Based on our definition of the lateral motion events, the ratio for left turns in the whole dataset is only 1.54% and that for the right turns is 1.49%. Even though the appearances of both events are rare, the rates are close, which makes common sense. In the following methodology section, multiple technics will be discussed about eliminating the negative influence of this dataset unbalance between a large number of no turns and a dramatically small quantity of turning events as compared with no turns.

### 3.3. Features Creation Based on Summary Statistics

Summary statistics are widely utilized in existing research [4,6] for analyzing time series data within the field of machine learning. They have an advantage in effectively reflecting the numerical characteristics of data. We adopted four summary statistics including mean, standard deviation, minimum, and maximum, as new elements of our features. Intuitively, the sudden variation of vehicles’ motion indicates the drivers’ intention to change the current driving status. When drivers feel comfortable about the present situation, they tend to keep speed and the vehicle remains stable. The larger the standard deviation and/or maximum values are, the greater the possibility that the driver is erratic. Based on effectiveness, we introduce four summary statistics including mean, standard deviation, minimum, and maximum, as new elements to our features.

Since there are d elements in each data chunk, the dimension of the summary statistics indices should be 4d. Therefore, we reshape the data chunk by concatenating the row vectors of original chunks and appending the row vector of summary statistics. The new data vector is shown in Equation (2).

(2) $$D^{\prime }{}^{\left(t\right)}=\left[\begin{array}{c} X_{t-n},X_{t-(n-1)},\cdots ,X_{t-m},S_{t} \end{array}\right]$$

where $X_{t^{\prime }}=[x_{t^{\prime }}^{\left(1\right)},\cdots ,x_{t^{\prime }}^{\left(d\right)}]$ is the row vector at time $t^{\prime }$ in the original matrix $D^{\left(t\right)}$; $S_{t}$ is the summary statistics vector as in Equation (3).

(3) $$S_{t}=\left[\begin{array}{c} Mean\left(D^{\left(t\right)}\right),Std\left(D^{\left(t\right)}\right),Min\left(D^{\left(t\right)}\right),Max\left(D^{\left(t\right)}\right) \end{array}\right]$$

where, the Mean, Std, Min, and Max row vectors are in the same shape. Take the Mean vector as an example in Equation (4).

(4) $$Mean\left(D^{\left(t\right)}\right)=\left[\begin{array}{c} \frac{\sum \left(Y^{\left(1\right)}\right)}{\left(t-m\right)-\left(t-n\right)+1},\cdots ,\frac{\sum \left(Y^{\left(d\right)}\right)}{\left(t-m\right)-\left(t-n\right)+1} \end{array}\right]$$

where, $Y^{\left(i\right)}=[x_{t-n}^{\left(i\right)},x_{t-(n-1)}^{\left(i\right)},\ldots ,x_{t-m}^{\left(i\right)}{]}^{T}$ is the i-th column of the original data chunk, i∈{1,…,d}.

### 3.4. Features Creation with Fast Fourier Transform

The fast Fourier transform (FFT) can transfer the data sequence from the time domain to the frequency domain in order to reveal the rate of recurrence characteristics of the sequence. Zhang et al., in 2010, utilized the FFT data to recognize the driving patterns of the drivers in [19], and set up an example to use frequency domain features to reveal the dynamic characteristics of the driver behaviors. Therefore, the FFT can also be useful in the current study to provide more frequency information than normal vehicle dynamic mobility data. Using the temporal data sequence $Y={\left[x_{1},\;x_{2},\;\ldots ,\;x_{N}\right]}^{T}$ as an example, the FFT coefficients of Y are shown in Equation (5).

(5) $$Y[n]=\sum_{k=1}^{N}x_{k}e^{-j\frac{2\pi }{N}nk},n=1,\ldots ,N$$

When a driver starts to make the attempt to turn or shift lane, they tend to manipulate the vehicle more. This makes the vehicle’s motion status and devices’ statuses vary more frequently than during normal driving (i.e., staying in the lane). Using the FFT method, we can analyze the temporal data sequence of each element in the data chunk, find out the frequency with the highest FFT coefficient, and observe the variation of said frequency which gives some indication of changing driving status. These frequencies of the elements are calculated and concatenated to the reshaped data vector as new features are created. Both groups of the created features, summary statistics, and FFT frequencies can be quickly calculated. The limited computing resources needed will allow data processing while driving.

### 3.5. Feature Selection

In order to make the application easy to be deployed in vehicles and increase the raw data availability, we focused on kinetic measurements, such as vehicle acceleration, and excluded several features that are either irrelevant, such as the number of GPS satellites and the headlight status, or directly indicated the turning action, such as crossing the left lane track and crossing the right lane track. The selected features are described in Table 1. It is to be noted that the “distance to left (right) marker” in the table indicates the distance from vehicle centerline to the inside of the left-side (right-side) lane marker. 

To illustrate the kinetic characteristics of the observations, we also included the first and the second derivatives of the “longitudinal acceleration” with respect to time as well as the speed and the acceleration of the vehicle to the left and right markers are also included. Take the jerk (first order time derivative of longitudinal acceleration) as an example. Using $\alpha_{acc}={\left[\begin{array}{cccc} x_{t-n}^{\left(5\right)}, & x_{t-(n-1)}^{\left(5\right)}, & \cdots , & x_{t-m}^{\left(5\right)} \end{array}\right]}^{T}$ to express the vector of longitudinal acceleration in the matrix $D^{\left(t\right)}$ of the original dataset, the jerk is calculated as shown in Equation (6).

(6) $$jerk={\left[\begin{array}{cccc} \left(x_{t-n}^{\left(5\right)}-x_{t-\left(n+1\right)}^{\left(5\right)}\right), & \left(x_{t-(n-1)}^{\left(5\right)}-x_{t-n}^{\left(5\right)}\right), & \cdots , & \left(x_{t-m}^{\left(5\right)}-x_{t-\left(m+1\right)}^{\left(5\right)}\right) \end{array}\right]}^{T}$$

The number “5” appears here and indicates the longitudinal acceleration is the fifth feature in the dataset. Other aforementioned features can be taken as similar discrete differentials and be calculated in the same manner.

Thus, there are 15 basic features. We included summary statistics, as well as FFT analysis of these features as other features. Here we used 5 s as the total time window for each lateral motion event. With m=5, this window resulted in 750 features.

**Figure 1:**
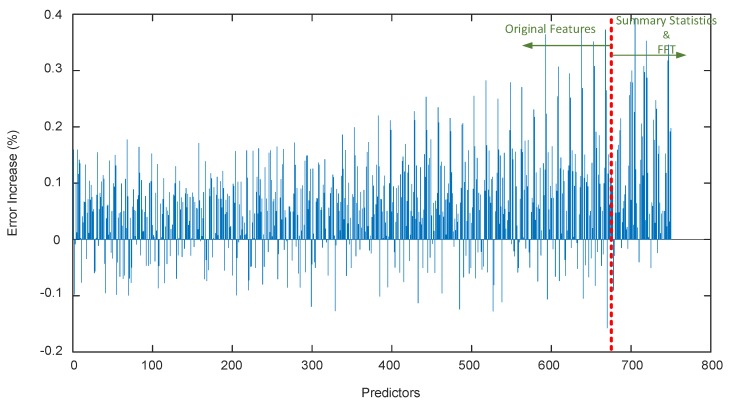
Random forest misclassification error increase after predictor random permutation.

The importance of prediction features was evaluated with random forest (RF) [20] and its corresponding predictor permutation technique [21]. RF is an ensemble classifier that is composed of decision tree classifiers that have been generated based on random portions of the training data observations and features. The RF was first trained on the training data. The features of the training data were then methodically permuted and fed back to the RF. The out-of-bag (OOB) error was then monitored on this new data. If the OOB error increased significantly, that feature was important in the classification process. The OOB errors for each feature are shown in Figure 1. The results show, intuitively, that features that occurred closer to the lateral motion event are more important in classification as they are more indicative of a lateral motion event. As can be seen, summary statistics and FFT magnitudes of the window are an effective processing step as they are generally more important than other features. 

**Figure 2:**
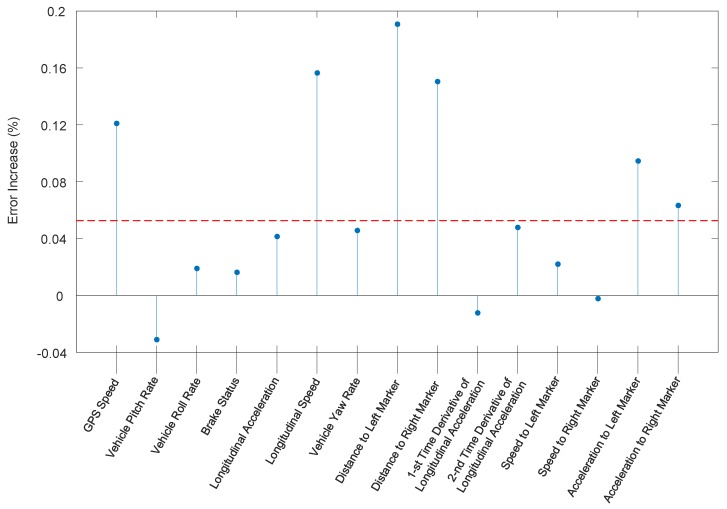
Mean of the importance of the original features.

Taking the feature vector and concatenating it back into the matrix form with one feature across time per column, we can average the error increase in each column to determine which measurements are the most important for lateral motion. As shown in Figure 2, the 1st, 6th, 8th, 9th, 14th, and 15th features are more important than the other nine features. Such outstanding features are the GPS speed (acquired by GPS), longitudinal speed (acquired by in-vehicle devices), distances to the left and right lane markers, and lateral acceleration of the vehicle. This is intuitively correct since vehicles lateral motions are highly relevant with their kinetic characteristics and relative positions to lane edges. Therefore, considering this relevance and easy accessibility, the original features described in Table 1 are selected.

## 4. Two-Stage Methodology

**Figure 3:**
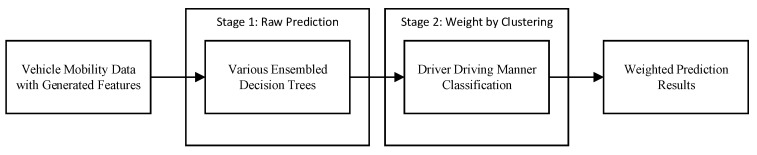
Two-stage method schematic diagram.

A two-stage data-driven method was designed to predict the lateral motion of the preceding vehicle based on the processed dataset in the previous section. The steps are shown in Figure 3. The vehicle mobility data processed in the previous section is taken as the input to the method. Two ensembled decision trees were trained to provide the initial lateral motion prediction results. Based on the driver behavior or driving manner classification, the initial results were given various weights and the combination of the weighted results were taken as the final prediction.

To be more specific, in this paper, we used one-day data of a two-month dataset to be the training dataset. Our training set contained mobility data of 51 unique vehicles within 587,812 time instances (16.33 h of driving) [5]. One bagging decision tree [22] and one random under-sampling (RUS) boosted decision tree [23] were trained on the dataset to predict lateral motion. A GMM was used to cluster drivers based on the erraticness of their driving [24]. The dataset for the clustering combined the training data and the testing instance. Generally, the model created a voting scheme between two ensembles weighted by the driver type probabilities in order. In this way, the model skewed the classification away from false negatives with more erratic drivers.

### 4.1. Ensembled Decision Tree Classification

Considering the skewness nature of the dataset, we conducted multiple experiments based on different classification methods (three times for each method), including the supporting vector machine (SVM), basic decision tree (DT), and several ensemble decision trees (EDT). The bagging decision trees and the RUS boosted decision trees demonstrated better performance on the cross-validations.

#### 4.1.1. Bagging Decision Trees

The term “bagging” is an acronym of “bootstrap aggregating” [22]. The learning scheme was created to improve the classification accuracy of the traditional algorithms by using a bootstrap [25] method to randomly sample the training dataset when training multiple weak learners, or decision trees in this instance, and to make a prediction based on the voting or average of the results of decision tree trained on the sampled datasets. Take $T=\{(l_{n},x_{n}),n\in \{1,\ldots ,N\}\}$ as the training dataset, where $l_{n}$ is the label or class of the n-th training data instance $x_{n}$. A normal DT is trained on all the training data to make future predictions of the testing data $x_{test}$ as shown in Equation (7).

(7) $$l_{test}=\psi \left(x_{test},T\right)$$

Within the bagging decision trees algorithm, bootstrap samples $\left\{T^{b}\right\}$ are repeatedly and randomly drawn, with replacement, from the original training dataset T when training each tree in the ensemble. The final prediction was then obtained as a vote among the DTs in the ensemble. For this work, the labels include {Turn Left, Won’t Turn, Turn Right}, i.e., {−1,0,1}.

The adaptive synthetic (ADASYN) sampling approach [26] was utilized to deal with the imbalanced training data. The algorithm generated more synthetic data for the minority classes which were Turn Left and Turn Right here in this case. We used ADASYN to generate the synthetic data for Turn Left class against the Won’t Turn class examples and that for Turn Right class against the Won’t Turn class examples, respectively. The combined dataset of these two new datasets formed the actual training dataset. Ten-fold cross-validation results of the bagging ensembled based on the training dataset processed by the ADASYN are shown in Table 2.

In this table, false negative rates are more than 43% for Turn Left and around 59% for Turn Right which was due to the high disparity between classes. On the other hand, the bagging trees ensemble tended to bias more conservatively towards a no turn prediction.

#### 4.1.2. Random Under-Sampling Boosted Decision Trees

The RUS boosted decision trees were utilized to resolve skewness of the training data. The idea was to down-sample the training dataset using RUS so that the amount of the minority class data was comparable to that of the majority class data. The ratio of the quantities of different classes was predefined to make the observations of the three classes equal to each other. The weak learners we used were normal decision trees. For each iteration, the under-sampling was conducted based on the predefined ratio, and the weight for each instance of the sampled training dataset was calculated. The sampled dataset and the corresponding weights were used to train one weak learner. According to the error, the weight for the current weak learner was computed and the weights of each instance were adjusted based on it. After predefined times of iteration, multiple weak learners were generated based on training datasets that had more class-balanced data. For each testing instance, the weighted voting of each weak learner classification results was the prediction. The RUS boosted trees ten-fold cross-validation results are shown in Table 3. In this table, the false negative rates have been largely improved. The RUS boosted trees performed effective prediction of lateral motion. It is to be noted that the false positive cases are important here. In order to decrease the positive predictive value, this method tends to be more aggressive with its positive (−1, 1) labels at the expense of the false positives increasing from 2.33% to 13.81% as compared with the bagging method.

### 4.2. Gaussian Mixture Models Clustering

The ensemble decision trees algorithms expressed two opposite biases on the prediction results. We observed such biases in several different learning schemes. To secure safer trips, we utilized these biases with predictions on more erratic drivers given that they have more of a tendency to change current driving status. Since the bagged trees tended to provide more conservative predictions, i.e., predicted a lack of lateral motion when it did occur (false negatives), it reduced risk if we chose to cast less trust on its results when facing more erratic drivers. At the same time, since the RUS boosted trees tended to provide more aggressive predictions, i.e., falsely predicting turns (false positives), it reduced risk if we choose to cast more trust on its results when facing front drivers driving with more erratic manners.

Multiple well-developed studies have been conducted to cluster the behaviors of drivers based on their aggressiveness. Kanarachos et al., in 2018, summarized the features used to implement the clustering, including acceleration and smoothness (variance of acceleration) [27]. The harsh acceleration and harsh cornering behavior of the drivers mentioned in [28] are widely used for driver behavior classification. Their connections with the vehicle lane changes are also revealed. Therefore, the relevant data in the current dataset were assessed and corresponding metrics were selected for the clustering.

**Figure 4:**
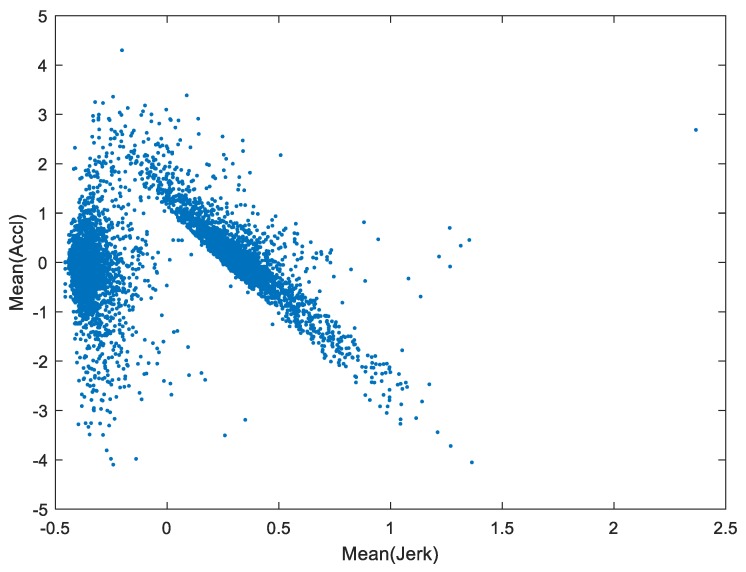
Original data grouping behavior.

As observed in the training dataset, the mobility data showed a fairly clear boundary between the two groups of drivers. We took data of two features, the mean of longitudinal acceleration and the mean of the jerk, as an example and showed the two-dimensional plot of them in Figure 4. It can be seen that there is a group of vehicles that have comparatively small or even negative mean values of jerk. On the other hand, the other group of vehicles, or more specifically their drivers, tend to use more variable and aggressive acceleration actions which is illustrated by the increment of acceleration (positive and high jerk). Given that the mean acceleration can be determined by different driving scenarios and environments, which can be encountered by any driver, it is more reasonable to take the driving manners with more frequent actions and a tendency to increase the acceleration as more erratic. Therefore, after analyzing the physical meaning of the data, we clustered the drivers into two categories: consistent (the former group) or erratic (the latter group), and this provided a theoretical basis for the further processing of the ensemble trees predictions. 

In order to combine the two algorithms’ ascendancy on the algorithm, we utilized GMM to cluster the driver into one of the two clusters. The clusters of GMM are represented by two different Gaussian distributions characterized by distinct expectations and variances. Here we use $N\left(\mu_{1},\;\Sigma_{1}\right)$ as the distribution for consistent drivers’ cluster and $N\left(\mu_{2},\;\Sigma_{2}\right)$ as the distribution for erratic drivers’ cluster. The clustering problem is thus transformed into finding the distributions of the observations in the dataset, which means to find the $\mu_{1}$, $\Sigma_{1}$, $\mu_{2}$, and $\Sigma_{2}$. 

**Figure 5:**
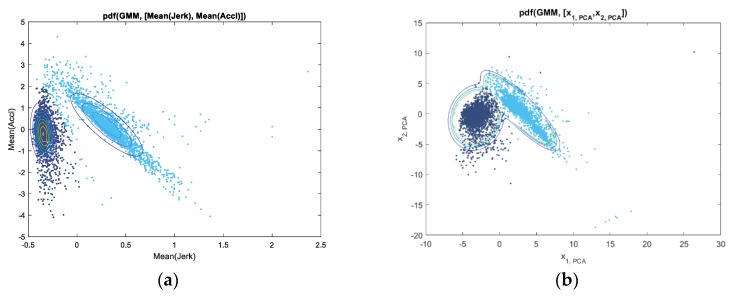
GMM clusters results based on (**a**) original data; (**b**) PCA processed data.

**Figure 6:**
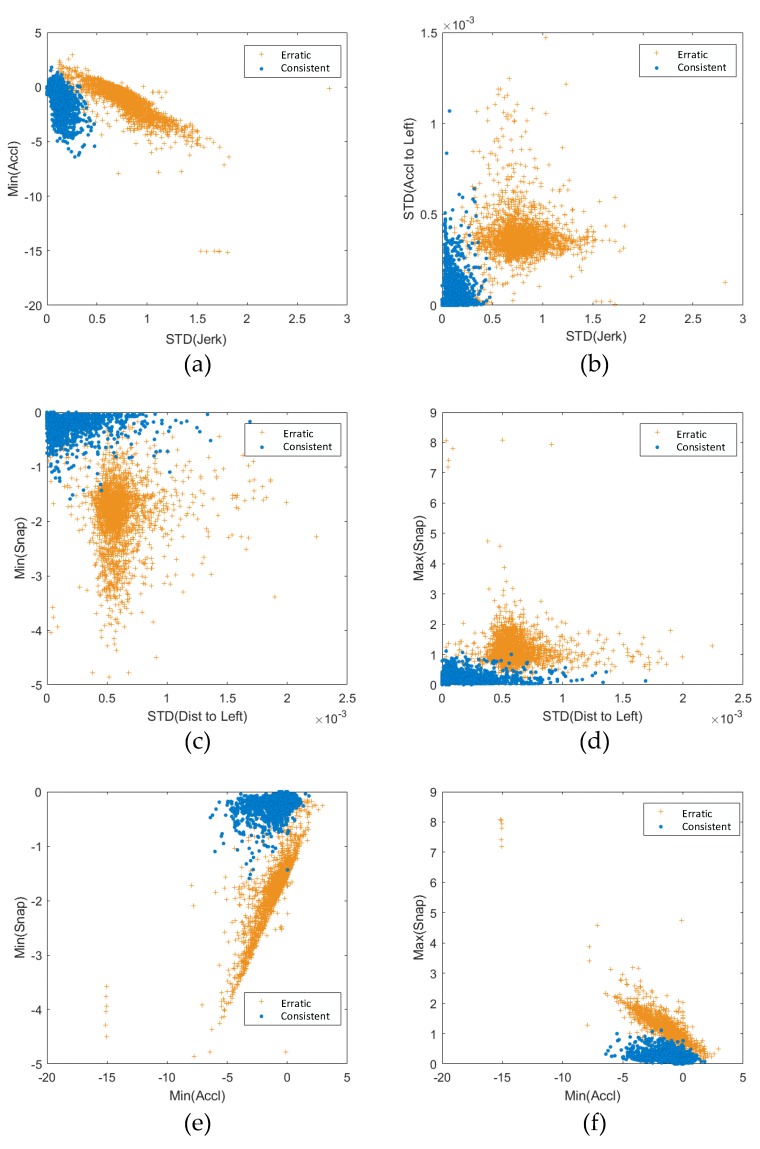
Kinematic behaviors grouping.

The clustering results based on the training samples are listed in Figure 5a,b. The GMM is conducted on the mean of the longitudinal acceleration versus the mean of the longitudinal jerk within the original training dataset in Figure 5a. We also used principal component analysis (PCA) to analyze the training data. The GMM was also conducted on the processed data after PCA, and this is shown in Figure 5b. Using the clustering model based on original data, we also plotted the grouping characteristics of multiple kinematic variables as shown in Figure 6. According to these plots, there exist two groups of driving behaviors, and based on the physical meaning of these kinematic variables, we categorize them into two classes: more consistent drivers and less consistent drivers.

The algorithm can provide two probabilities, i.e., $p_{1}^{\left(i\right)}$ and $p_{2}^{\left(i\right)}$ that indicate the chances the driver belongs to each of the two clusters or distributions. Since the two posteriors indicate the probabilities of the driver belonging to a respective cluster, and they sum to one, they are naturally suitable for normalizing the two predictions made by the ensembled methods to find a new average. Take $\alpha =p_{1}^{\left(i\right)}$ to be the probability of the front driver belongs to the consistent drivers’ cluster and $\beta =p_{2}^{\left(i\right)}$ to be that of the front driver belongs to the erratic drivers’ cluster. The final prediction is shown in Equation (8).

(8) $$C_{F}=quant\left(\begin{array}{cc} \alpha C_{Bagging}+\beta C_{RUSBoosted}, & 1 \end{array}\right)$$

where, $C_{F}$ is the final prediction; $C_{Bagging}$ and $C_{RUSBoosted}$ are the predictions made by the bagging DTs and the RUS boosted DTs, respectively; the quant(⋅, 1) is the quantize function that categorizes the real number into the set of {−1, 0, 1}, which means turn left, won’t turn, and turn right. 

**Figure 7:**
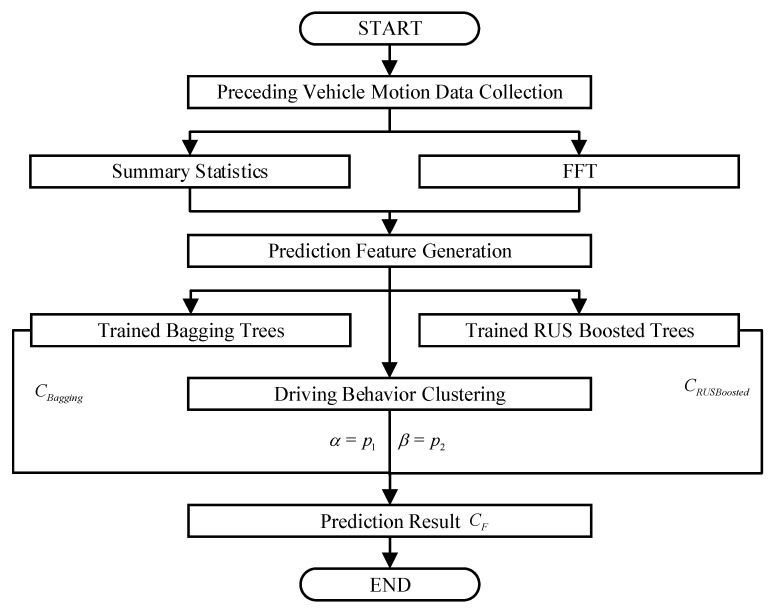
Two-stage method flow chart.

The flow chart of the two-stage method is shown in Figure 7.

## 5. Experiments Results and Discussion

The remaining 60-day observation data in the aforementioned two-month dataset was used as test data for the demonstration of the effectiveness of the proposed prediction algorithm. The dataset collected on April 11, 2013 and used as training data in the previous methodology was excluded. 

After the training process, 40% of the data were used for the model test. The prediction results of the trained bagging trees are shown in Table 4. The false-negative rate is around 36.35%, and the false-positive rate is around 4.37% which is less meaningful, because of the large quantity of the “won’t turn” events. The prediction results using trained RUS boosted trees are shown in Table 5. The false-negative rate has largely decreased here, and it is 5.37%. The false-positive rate is 11.91%.

Using the proposed GMM clustering integrated algorithm, the prediction results are shown in Table 6 (without PCA) and Table 7 (with PCA). 

As demonstrated in Table 4, Table 5, Table 6 and Table 7, in the scenarios where the GMM algorithm was integrated, with or without PCA, and were utilized, the prediction results perform more balanced than that of either one of the two ensemble methods. The false-negative rates are notably smaller than the bagging trees and the false-positive rates are also remarkably smaller than the RUS boosted trees while the true-positive and true-negative rates are not remarkably sacrificed. The comparison demonstrated the effectiveness and advantages of the GMM clustering serving as a preprocessing step before the actual prediction procedure. As for the PCA processing, it can easily be seen that this process largely increases the true-positive rate, and decreases the false-negative rate, and makes the prediction more accurate. The false-positive rate is also influenced and downgrades the accuracy. However, in general, the algorithm with PCA can make the results more conservative by predicting more active motions of the vehicle. Thus, in reality, this “preference” of the prediction algorithm could provide more safety. 

For comparison, the observations with the 0.1 s prediction time were also generated. The results of the implementation based on this dataset are listed in the following tables. The prediction results of the trained bagging trees are shown in Table 8. The false-negative rate has decreased to 21.09% and the false-positive rate has also decreased to 3.10%. The prediction results using trained RUS boosted trees are shown in Table 9. The false-negative rate almost remains the same, and it is 5.68%. The false-positive rate is 10.51%.

Using the proposed GMM clustering integrated algorithm, the prediction results are shown in Table 10 (without PCA) and Table 11 (with PCA). One can see notable improvement of the accuracy with a shorter prediction time, i.e., 0.1 s. The false-negative rate decreased from 28.95 to 15.94% without PCA, and from 10.14 to 8.14% with PCA. The false-positive rate also decreased from 6.12 to 4.76% without PCA, and from 10.76 to 9.52% with PCA. The results coincide with our intuition that the closer to the actual vehicle actions the more accurate is the prediction. 

In addition, we observed skewness with the 0.5 s prediction time. It seems that the prediction on the motion towards the right is more accurate than the others in terms of the false-negative rate. While with the corresponding false-positive cases considered, the trained decisions trees tend to provide more “turn right” predictions. The reason could be that in the training dataset, there are more “turn right” events. 

Overall, the high accuracy of the prediction results indicates that the proposed algorithm is effective and the idea of predicting the motion of other vehicles based on their accessible mobility data is realistic. Though some of the accuracy parameters can reach a level of more than 95%, the absolute numbers of the misclassifications are still to be decreased. This problem could be resolved with larger and more diverse training dataset, more detailed clusters, and even shorter prediction time. Because the functionality can be used in the autonomous driving vehicles, the computing capability of the vehicle-installed computers and processing capability of the corresponding software systems can tolerate much shorter reaction time than 0.1 s. The deployment of the proposed algorithm on such vehicles can downgrade much on-road uncertainty and provide more safety for the passengers. 

## 6. Conclusions

In this paper, we proposed an algorithm to predict the preceding vehicles’ lateral motions with their real-time mobility data considering the classification of drivers’ behaviors. The lateral motion prediction functionality can serve as part of the autonomous vehicle driving systems or other driver assistance software. The data-drive prediction technique includes two parts: drivers’ behaviors clustering and ensembled decision trees (bagging and RUS boosted) prediction. We validated the technique with the real-world vehicle mobility data from the SPMD database and the random forest feature selection. We also used summary statistics and the FFT to process the time series data. The prediction results on the real-world vehicle data demonstrate the effectiveness of the algorithm and its potential to be utilized in on-road vehicles. With more available complete and diverse real-world vehicle mobility data, the implementation of the prediction system can have higher accuracy and thus provide the driving assistant systems with more useful driving information. In the context of the emerging autonomous driving technique, it is expected to experience a time period when both autonomous driving systems and human drivers coexist. The application of the proposed prediction technique can decrease the uncertainty brought by human drivers in preceding cars, which can improve the safety level of drivers and passengers in vehicles as well as pedestrians. The designed approach also contributes to the development of highly automated transportation systems as well as building intelligent and smart cities.

## Figures and Tables

**Table 1:** Selected features from the original dataset.

Element Name	Units
GPS speed	meters/second
Vehicle pitch rate	degrees/second
Vehicle roll rate	degrees/second
Brake status	none
Longitudinal acceleration	meters/second^2^
Longitudinal speed	meters/second
Vehicle yaw rate	degrees/second
Distance to left marker	millimeter
Distance to right marker	millimeter

**Table 2:** The confusion matrix of the bagging trees.

Bagging Trees		Predicted Class		
		Turn Left	Won’t Turn	Turn Right
**True Classes**	Turn left	88	67	0
	Won’t turn	78	5324	49
	Turn right	2	66	46

**Table 3:** The confusion matrix of the RUS boosted trees.

RUS Boosted Trees		Predicted Class		
		Turn Left	Won’t Turn	Turn Right
**True Classes**	Turn left	149	4	2
	Won’t turn	384	4698	369
	Turn right	4	6	104

**Table 4:** The confusion matrix of the bagging trees trained by 61-day data.

Bagging Trees		Predicted Class		
		Turn Left	Won’t Turn	Turn Right
**True Classes**	Turn left	1392	1165	10
	Won’t turn	1726	154,939	5346
	Turn right	9	651	1821

**Table 5:** The confusion matrix of the RUS boosted trees trained by 61-day data.

RUS Boosted Trees		Predicted Class		
		Turn Left	Won’t Turn	Turn Right
**True Classes**	Turn left	2400	104	63
	Won’t turn	9944	142,722	9345
	Turn right	60	44	2377

**Table 6:** The confusion matrix of the proposed method without PCA.

Proposed Method Without PCA		Predicted Class		
		Turn Left	Won’t Turn	Turn Right
**True Classes**	Turn left	1614	884	22
	Won’t turn	3437	152,102	6472
	Turn right	20	505	1898

**Table 7:** The confusion matrix of the proposed method with PCA.

Proposed Method with PCA		Predicted Class		
		Turn Left	Won’t Turn	Turn Right
**True Classes**	Turn left	2212	258	50
	Won’t turn	8761	144,577	8673
	Turn right	47	146	2230

**Table 8:** The confusion matrix of the bagging trees with 0.1 s prediction time.

Bagging Trees		Predicted Class		
		Turn Left	Won’t Turn	Turn Right
**True Classes**	Turn left	1989	470	13
	Won’t turn	2409	156,959	2618
	Turn right	21	566	2014

**Table 9:** The confusion matrix of the RUS boosted trees with 0.1 s prediction time.

RUS Boosted Trees		Predicted Class		
		Turn Left	Won’t Turn	Turn Right
**True Classes**	Turn left	2313	111	48
	Won’t turn	7212	144,957	9817
	Turn right	74	55	2472

**Table 10:** The confusion matrix of the proposed method without PCA with 0.1 s prediction time.

Proposed Method without PCA		Predicted Class		
		Turn Left	Won’t Turn	Turn Right
**True Classes**	Turn left	2071	337	21
	Won’t turn	3524	154,268	4194
	Turn right	35	400	2110

**Table 11:** The confusion matrix of the proposed method with PCA with 0.1 s prediction time.

Proposed Method with PCA		Predicted Class		
		Turn Left	Won’t Turn	Turn Right
**True Classes**	Turn left	2219	170	40
	Won’t turn	6528	146,563	8895
	Turn right	66	129	2350
